# Glucose-6-phosphate dehydrogenase correlates with tumor immune activity and programmed death ligand-1 expression in Merkel cell carcinoma

**DOI:** 10.1136/jitc-2020-001679

**Published:** 2020-12-23

**Authors:** Motoki Nakamura, Kotaro Nagase, Maki Yoshimitsu, Tetsuya Magara, Yuka Nojiri, Hiroshi Kato, Tadahiro Kobayashi, Yukiko Teramoto, Masahito Yasuda, Hidefumi Wada, Toshiyuki Ozawa, Yukie Umemori, Dai Ogata, Akimichi Morita

**Affiliations:** 1 Departments of Geriatric and Environmental Dermatology, Nagoya City University Graduate School of Medical Sciences, Nagoya, Japan; 2 Division of Dermatology, Department of Internal Medicine, Faculty of Medicine, Saga University, Saga, Japan; 3 Department of Molecular Pathology of Skin, Faculty of Medicine, Kanazawa University, Kanazawa, Japan; 4 Department of Skin Oncology/Dermatology, Saitama Medical University International Medical Center, Hidaka, Japan; 5 Department of Dermatology, Gunma University, Maebashi, Japan; 6 Environmental Immuno-Dermatology, Yokohama City University, Yokohama, Japan; 7 Department of Dermatology, Osaka City University, Osaka, Japan; 8 Division of Dermatology, Nagaoka Red Cross Hospital, Nagaoka, Japan; 9 Department of Dermatology, Saitama Medical University, Iruma-gun, Japan

**Keywords:** skin neoplasms, tumor biomarkers, immunotherapy, programmed cell death 1 receptor

## Abstract

**Background:**

Merkel cell carcinoma (MCC) is a rare and highly malignant skin cancer. Some cases have a good prognosis and spontaneous regression can occur. Reported prognostic markers, such as Merkel cell polyoma virus infection or programmed death ligand-1 (PD-L1) expression, remain insufficient for precisely estimating the vastly different patient outcomes. We performed RNA sequencing to evaluate the immune response and comprehensively estimate prognostic values of immunogenic factors in patients with MCC.

**Methods:**

We collected 90 specimens from 71 patients and 53 blood serum samples from 21 patients with MCC at 10 facilities. The mRNA was extracted from formalin-fixed paraffin-embedded tissues. Next-generation sequencing, immunohistochemical staining and blood serum tests were performed.

**Results:**

Next-generation sequencing results classified MCC samples into two types: the ‘immune active type’ was associated with better clinical outcomes than the ‘cell division type’. Expression of the glucose-6-phosphate dehydrogenase (G6PD) gene was highly significantly upregulated in the ‘cell division type’. Among 395 genes, G6PD expression correlated with the presence of lymph node or distant metastases during the disease course and significantly negatively correlated with PD-L1 expression. Immunohistochemical staining of G6PD also correlated with disease-specific survival and exhibited less heterogeneity compared with PD-L1 expression. G6PD activity could be measured by a blood serum test. The detection values significantly increased as the cancer stage progressed and significantly decreased after treatment.

**Conclusions:**

G6PD expression was an immunohistochemically and serum-detectable prognostic marker that negatively correlated with immune activity and PD-L1 levels, and could be used to predict the immunotherapy response.

## Background

Merkel cell carcinoma (MCC) is a rare but highly malignant skin cancer. The reported prognosis is poor, with a 5-year survival rate of 0%–18%.^1^ Some cases, however, have a good prognosis, and spontaneous regression after biopsy can occur. The frequency of spontaneous regression in MCC is 1.7%–3.0%.^2^ This ratio is much higher than that for other solid carcinomas. Immune responses, such as T-cell-mediated immunity, might be related to tumor regression and some types of MCC may have high sensitivity to an immune response. We previously reported that increased expression of programmed death ligand 1 (PD-L1) in metastatic MCC lesions strongly correlates with a better clinical outcome.^3^ PD-L1 is an immunoinhibitory molecule that suppresses T cell activation. PD-L1 upregulation in cancer cells typically indicates the evasion of antitumor immunity, but this relation between prognosis and PD-L1 expression in MCC is opposite that in other carcinomas. High PD-L1 expression seems to result from the activation of anti-tumor immunity in MCC and has, therefore, been reported as a prognostic marker.^4^ PD-L1 expression, however, may be heterogeneous, even in the same case.^5 6^ Although PD-L1 expression reflects the immune status at the time of evaluation, it is still difficult to predict a patient’s outcome on the basis of PD-L1 expression. Therapies involving blockade of immune checkpoints, including PD-L1 and its receptor programmed cell death 1 (PD-1), are producing successful results for MCC.^7 8^ Although PD-L1 expression is considered a potential predictive biomarker for sensitivity to immune checkpoint blockade, it has become clear that the potential for prediction on the basis of PD-L1 expression is limited^9^ because of its heterogeneity. Here, we performed RNA sequencing to evaluate the immune response and comprehensively estimate the prognostic values of immunogenic factors as potential predictive biomarkers for immunotherapy in MCC.

## Methods

### Study design and participants

A total of 90 formalin-fixed paraffin embedded (FFPE) samples from 71 Japanese patients with histologically diagnosed MCC on the basis of biopsy or surgical resection samples obtained at nine facilities were collected as previously reported.^3^ The cohort is summarized in online supplemental table 1. Immunohistochemical analyzes were performed on these samples. Of these 90 FFPE samples, 44 samples were randomly selected for RNA sequencing using a next-generation sequencer (NGS). Three samples were dropped from the study because of low gene expression and 41 samples were used for further analyzes (summarized in table 1). Blood serum samples were collected from another cohort of patients diagnosed with MCC at three facilities, and are summarized in table 2.

10.1136/jitc-2020-001679.supp1 Supplementary data

**Table 1:** Characteristics and treatment data for patents in NGS analysis

RNA for NGS		
Characteristics		Value
Cases		37
Samples		41
Age (range)		76.57 (40–98)
Sex	Male	13 (35.1%)
	Female	24 (64.9%)
Race	Asian (Japanese)	37 (100%)
Primary site		Cases(n=37)
	Head and neck	25 (67.6%)
	Trunk	1 (2.7%)
	Limbs	11 (29.7%)
Lesion		Samples(n=41)
	Primary	33 (80.5%)
	Skin meta	8 (19.5%)
Stage at collection		Samples (n=41)
	Ⅰ	13 (31.7%)
	Ⅱ	15 (36.6%)
	Ⅲ	8 (19.5%)
	Ⅳ	5 (12.2%)
Treatment		Cases (n=37)
	Surgery	7 (18.9%)
	RT	3 (8.10%)
	Surgery+RT	19 (51.4%)
	Surgery+chemo	2 (0.6%)
	Surgery+RT+chemo	2 (0.6%)
	Surgery+RT+ ICI	2 (0.6%)
	Observation	2 (0.6%)

ICI, immune checkpoint inhibitor; NGS, next-generation sequencer; RT, radiation therapy.

**Table 2:** Characteristics and treatment data for patients in blood serum tests

Blood serum		
Characteristics		Value
Cases		21
Samples		53
Age (range)		81.05 (62–97)
Sex	Male	10 (47.6%)
	Female	11 (52.4%)
Race	Asian (Japanese)	21 (100%)
Stage at collection		Samples (n=53)
	Ⅰ	25 (47.2%)
	Ⅱ	15 (28.3%)
	Ⅲ	8 (15.1%)
	Ⅳ	5 (9.4%)
Treatment		Cases (n=21)
	Surgery	8 (38.1%)
	Surgery+RT	10 (47.6%)
	Surgery+RT+ ICI	2 (9.5%)

Total percentage values might sum to >100% due to rounding.

Chemo, chemotherapy; ICI, immune checkpoint inhibitor; Meta, metastasis; RT, radiation therapy.

### RNA extraction and sequencing

Tumor tissue was carefully dissected from 3 to 5 undyed FFPE tissue sections (4 µm thickness) using a scalpel blade and deparaffinized in 640 µL deparaffinization solution (Qiagen, Hilden, Germany). Total RNA was refined using an AllPrep DNA/RNA FFPE Kit (Qiagen) according to the supplier’s instructions. The RNA integrity number and DV_200_ values were measured using a Bioanalyzer (Agilent Technologies, Santa Clara, California, USA) to evaluate the quality of the extracted RNA. RNA samples confirmed to be of sufficient quality were reverse-transcribed to cDNA using a SuperScript VILO cDNA Synthesis Kit (Thermo Fisher Scientific, Waltham, Massachusetts, USA) after assessing the density using a Qubit 4 Fluorometer (Thermo Fisher Scientific). cDNA samples were amplified and applied to the NGS using a PTC-100 thermal cycler (MJ Research, Watertown, Massachusetts, USA) and Ampliseq for the Illumina Immune Response Panel (Illumina, San Diego, California, USA). After quantification of the library using a Bioanalyzer, NGS analysis was performed using the MiniSeq System (Illumina). Data were uploaded and analyzed on the cloud-based software application BaseSpace Sequence Hub (Illumina). All data were uploaded to the national center for biotechnology information gene expression omnibus database (GSE154938).

### Gene set enrichment analysis

Gene set enrichment analysis (GSEA) was performed using the c5 Gene Ontology gene set collections as supplied by the Molecular Signatures Database^10^ and GSEA software (https://www.gsea-msigdb.org/gsea/).^11^

### Immunohistochemistry

Undyed FFPE tissue slides (Nunc, Roskilde, Denmark) were processed for indirect immunofluorescence to detect the expression of signal transduction proteins using primary antibodies to the anti-PD-L1 antibody (1:100, ab205921, Abcam, Cambridge, UK) as previously described.^3^ Bound antibodies were visualized with the appropriate secondary antibodies (Alexa Fluor 594 goat anti-rabbit IgG, A11005; Invitrogen, Waltham, Massachusetts, USA) at 37°C for 30 min at 1:100 dilution with 5% goat serum. 4’,6-diamidino-2-phenylindol (Vector Laboratories, Burlingame, California, USA) was used as a counterstain. The red fluorescence produced by Alexa 594 and blue fluorescence produced by 4’,6-diamidino-2-phenylindol were observed and captured using a fluorescence microscope BZ-X800 (Keyence, Osaka, Japan). The fluorescence intensities of PD-L1 were calculated using ImageJ Software (NIH, Bethesda, Maryland, USA) from 10 randomly selected fields as previously described.^3 5^ The pixel value of more than 45 was defined as ‘PD-L1 high’ and less than 35 was defined as ‘PD-L1 low’. Immunostaining for Merkel cell polyoma virus (MCPyV) was performed with large T-antigen (CM2B4 antibody; Santa Cruz Biotechnology, Santa Cruz, California, USA). Our cases exhibited a 69% positivity rate. The prognostic values of PD-L1 expression and MCPyV infection were analyzed in our previous work using the same cohort.^3^ Immunohistochemical staining of glucose-6-phosphate dehydrogenase (G6PD) was performed using anti-G6PD Rabbit IgG (1:25, HPA000247, MilliporeSigma, St. Louis, Missouri, USA) and a DAB substrate kit (SK-4100, Vector). The positive cells were counted using BZ-X800 from 10 randomly selected fields. A mean positive ratio of 50% or more was defined as high, and a mean positive ratio of less than 50% was defined as low.

### G6PD activity assay

G6PD activity was measured from blood serum using a G6PD activity assay kit (ab176722, Abcam) according to the supplier’s instructions. Fluorescence was monitored on a fluorescence microplate reader (Spectra Max Gemini EM, Molecular Devices, San Jose, California, USA) in kinetic mode for 40 min.

### Statistical analysis

NGS data were analyzed on the cloud-based software BaseSpace Sequence Hub (Illumina) using the RNA Amplicon application. A clustered heatmap of all samples was generated using the online tool iDEP.91 (http://bioinfomatics.sdstate.edu/idep/). Disease-specific survival was analyzed using the Kaplan-Meier method and log-rank test. The Kruskal-Wallis test was used to compare stages and a paired-t test was used to compare pretreatment and post-treatment in blood serum tests.

## Results

### RNA sequencing divided MCC samples into two types

**Figure 1:**
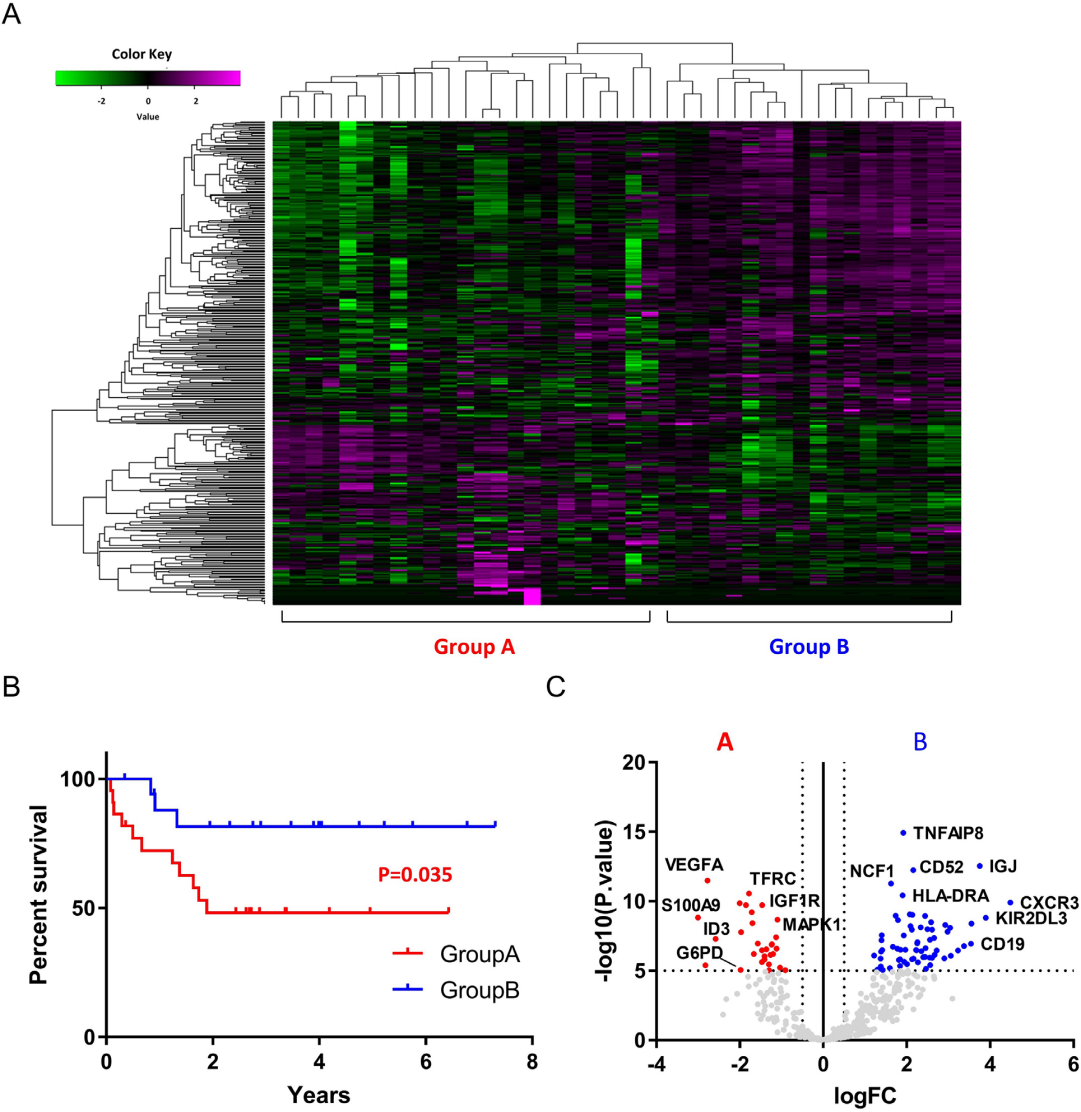
Gene expression heatmap of all 41 samples of MCC. Samples are divided into two groups based on the expression of 395 genes. Group A comprised 23 samples and group B comprised 18 samples (A). Kaplan-Meier survival analysis showed a significantly poorer prognosis for patients in group A compared with patients in group B (p=0.035, log-rank test) (B). Gene expression levels in each group are presented as volcano plots. Vertical and horizontal broken lines represent threshold of log2 fold-change (−0.5 and +0.5) and p value (1.0×10^–5^) (C). MCC, merkel cell carcinoma.

Total RNA was extracted from 44 samples and analyzed with NGS-targeted expression of 395 cancer biomarkers involved in tumor-immune system interactions and indicative of an immunotherapy response. Three samples were excluded due to low expression of all genes evaluated. Data from 41 samples were further analyzed. The characteristics of the 41 samples are summarized in table 1. The cohort included 13 men and 24 women with a median age of 76.57 (range 40–98) years. The most commonly affected site was the head and neck (25 samples, 67.6%), followed by the limbs (11 samples, 29.7%) and trunk (1 sample, 2.8%). Four samples (9.8%) showed spontaneous regression after biopsy. The mean RNA integrity number was 2.08 (1.0–2.6) and the mean DV_200_ value was 53.0 (28–88). Hierarchical cluster analysis was conducted with the 41 samples and the samples were divided into two groups according to the expression pattern of all 395 genes evaluated (figure 1A). Group A comprised 23 samples and group B comprised 18 samples. Kaplan-Meier survival analysis revealed a poorer prognosis for patients in group A compared with patients in group B (p=0.035, log-rank test, figure 1B). Gene expression levels in each group are presented as volcano plots in figure 1C. Vertical and horizontal broken lines represent threshold of log2 fold change (−0.5 and +0.5) and p value (1.0×10^–5^).

### Functional enrichment analysis of the gene expression in each type

**Figure 2:**
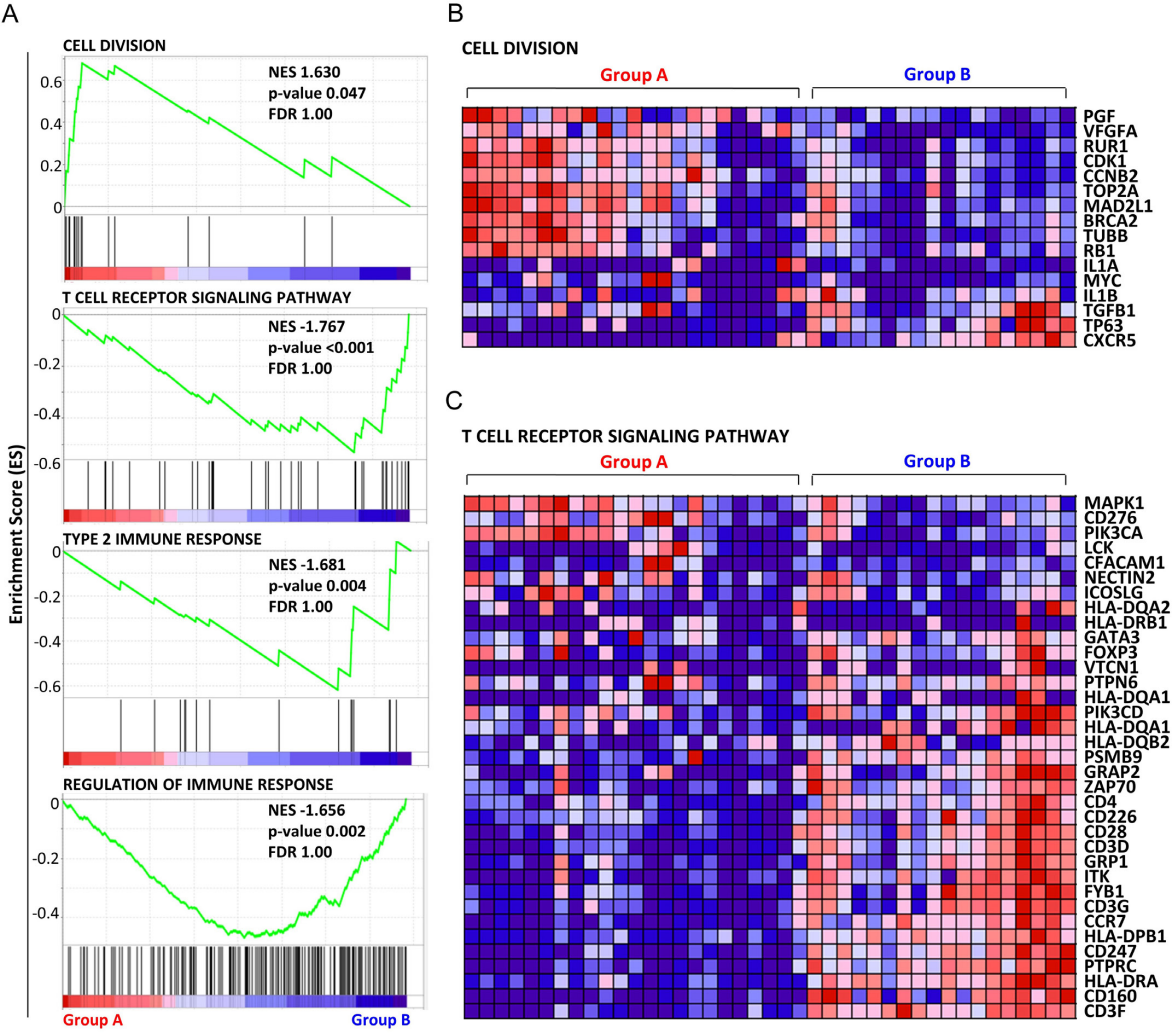
The top graph is the highest ranked and only one gene set had a p<0.05 in group A. The bottom three graphs are the top three ranked gene sets with smallest normalized enrichment scores (high relative expression in the group (B) with a p <0.01. Graphs display the enrichment score (y axis) versus the gene rank in an ordered dataset (x axis); genes with high relative expression in group A were given a low rank order value (leftmost tail), and genes with high relative expression in group B were given a high rank order value (rightmost tail) of the gene rank representation. Normalized enrichment scores, p values and FDR q values for each analysis are shown (A). Heatmap of analyzed genes constituting the top-ranked gene set for group A, ‘cell division’ (B). Heatmap of analyzed genes constituting the top-ranked gene set for group B, ‘T cell receptor signaling pathway’ (C). FDR, false discovery rate.

To interpret the classes of genes that were upregulated or downregulated in each group, GSEA was performed using the Gene Ontology resource including 10, 192 gene sets. The highest ranked and only gene set enriched in group A with a p<0.05 was the ‘cell division’ set. The normalized enrichment score was 1.63, and the p value was 0.047. In group B, we detected 49 gene sets with a p<0.05, and 7 gene sets with a p<0.01 (see online supplemental table 2). No gene sets with a false discovery rate (FDR) less than 0.25 were detected. Enrichment plots of representative gene sets in each group are shown in figure 2A. Graphs display the enrichment score (y axis) versus the gene rank in an ordered dataset (x axis); genes with high relative expression in group A were given a low rank order value (leftmost tail), and genes with high relative expression in group B were given a high rank order value (rightmost tail) of the gene rank representation. The rank of each gene in the respective gene set is indicated by the horizontal line below the enrichment plot. Normalized enrichment scores, p values and FDR q values for each analysis are shown. Gene expression heatmaps of the highest ranked gene sets for each group are presented in figure 2B. On the basis of these results, the two groups could be annotated as the ‘cell division type (group A)’ and the ‘immune active type (group B)’.

10.1136/jitc-2020-001679.supp2 Supplementary data

### G6PD is an indicator for classifying two types of MCC based on the tumor immune activity

**Figure 3:**
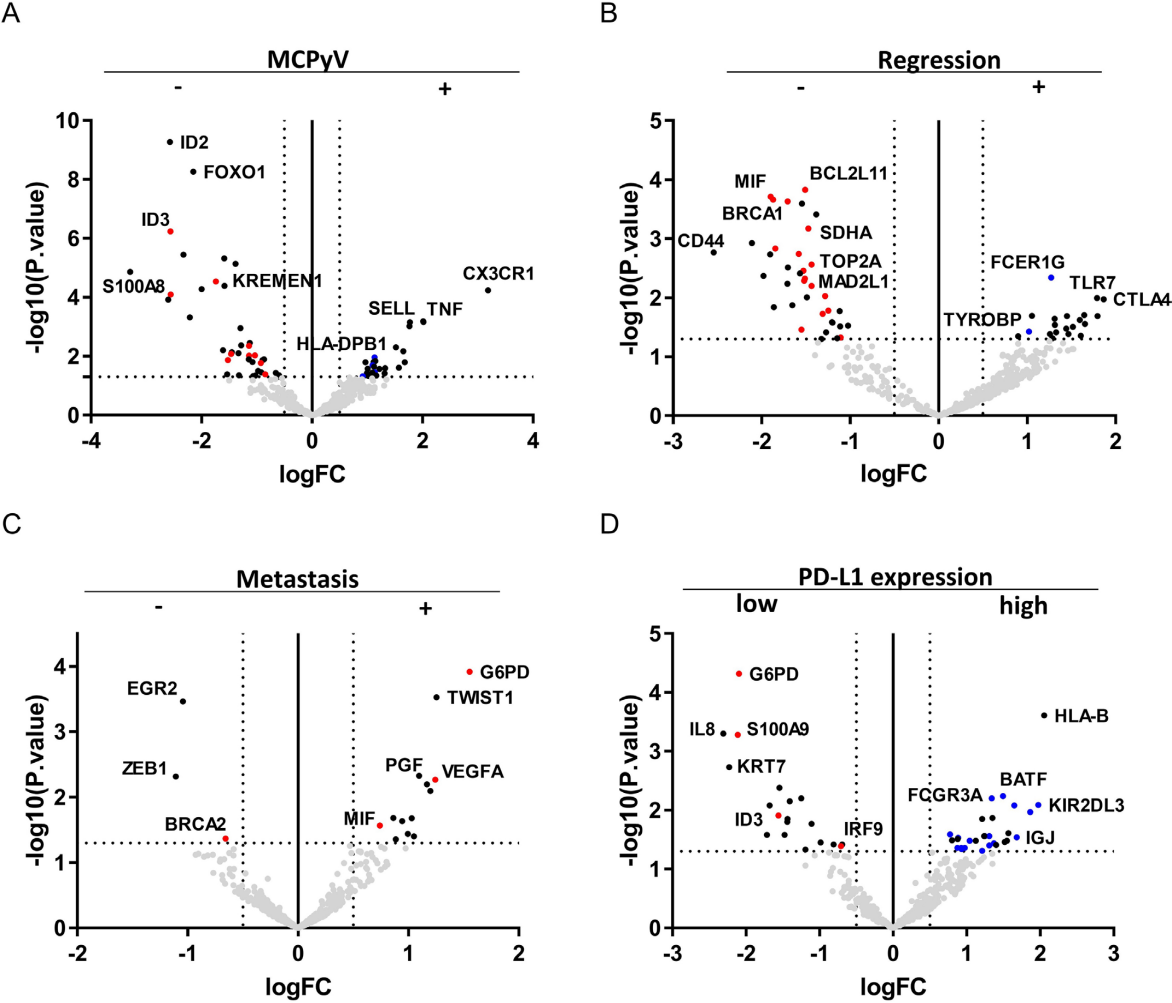
Merkel cell polyoma virus (MCPyV) infection, positive (n=24) versus negative (n=10) (A). Spontaneous regression after biopsy, positive (n=4) versus negative (n=29) (B). Lymph nodes or distant metastases during follow-up, positive (n=17) vs negative (n=17) (C). Programmed death ligand 1 (PD-L1) expression in tumor cells, high (n=18) vs low (n=10) (D). Red dots indicate significantly upregulated genes with p value <1.0×10^-5^ in group A ‘cell division type’ and blue dots indicate significantly upregulated genes with p value <1.0×10^-5^ in group B ‘immune active type’. The vertical and horizontal broken lines represent threshold of log2 fold-change (−0.5 and +0.5) and p value (0.05).

Significant differences in the gene expression between MCPyV infection positive (n=24) vs negative (n=10) (figure 3A), spontaneous regression after biopsy positive (n=4) negative (n=29) (figure 3B), cases have lymph node or distant metastasis during the follow-up positive (n=17) negative (n=17) (figure 3C), and PD-L1 expression in tumor cells high (n=10) vs low (n=18) (figure 3D) were analyzed. Red dots indicate significantly upregulated genes with p value <1.0×10^−5^ in group A ‘cell division type’ and blue dots indicate significantly upregulated genes with p value <1.0×10^−5^ in group B ‘immune active type’. Fisher’s exact tests revealed a significant association between groups A and B with regard to PD-L1 expression in MCC cells (p=0.032, OR=7.0, 95% CI 1.18 to 41.36, (see online supplemental table 3). High expression of PD-L1 is one factor of the upregulated immune activity observed in group B. High PD-L1 expression in MCC is associated with better clinical outcomes.^4^ PD-L1 expression is heterogeneous, however, even in the same case.^5 6^ This heterogeneity complicates the usage of this immune factor as a prognostic indicator. Actually, a previous immunohistochemical analysis using the same 90 samples revealed no significant correlation between PD-L1 expression in primary MCC lesions and clinical outcomes. PD-L1 expression correlates with patient prognosis only in skin metastatic lesions.^3^ We focused on G6PD as a substitute prognostic factor for PD-L1. G6PD was one of the highly and significantly upregulated genes in group A ‘cell division type’ (log2FC=−1.98, p=8.65×10^−6^, FDR=3.25×10^−5^, figure 1C). G6PD expression positively correlated with lymph node or distant metastasis during follow-up (log2FC=1.55, -value=1.22×10^−4^, FDR=0.020, figure 3C) and negatively correlated with PD-L1 expression (log2FC=−2.10, p value=4.84×10^−5^, FDR=0.017, figure 3D) and had the smallest p value among the 395 genes in both analyzes. G6PD can be used as an indicator for classifying 2 types of MCC and negatively correlates with PD-L1 expression and prognosis.

10.1136/jitc-2020-001679.supp3 Supplementary data

### Immunohistochemical expression of G6PD is a more useful prognostic predictor than PD-L1 expression

**Figure 4:**
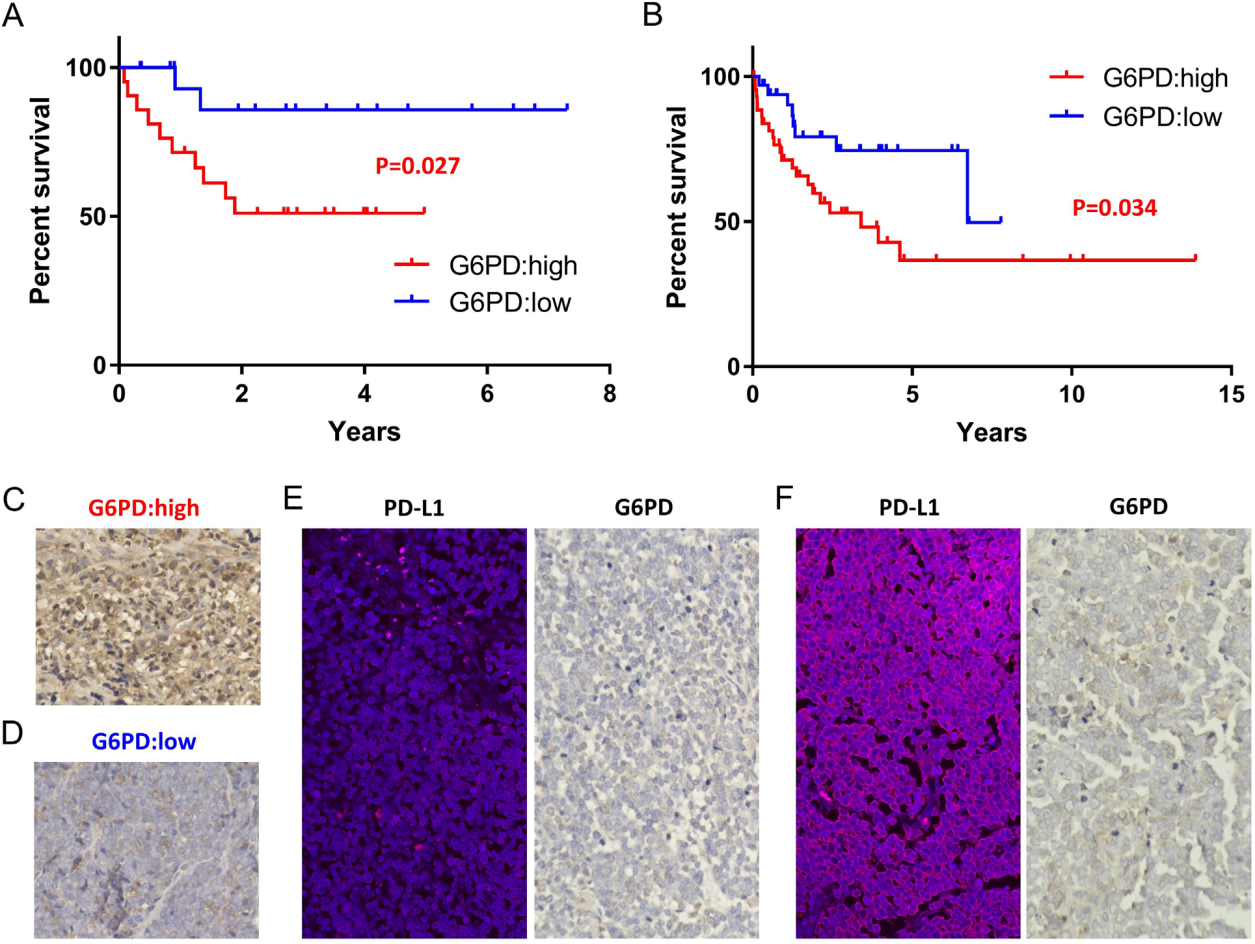
Kaplan-Meier survival curves comparing MCC with high and low G6PD expression classified on the basis of RNA expression (counts per million; CPM) using log-rank test (p=0.027, n=40) (A). Kaplan-Meier survival curves comparing MCC with high and low G6PD expression classified on the basis of immunohistochemistry using log-rank test (p=0.034, n=79) (B). Representative immunohistochemical staining in samples with high G6PD expression. This case showed spontaneous regression after biopsy and recurrent distant metastases after 10 months (C). Representative immunohistochemical staining samples with low G6PD expression. This case showed spontaneous regression after biopsy and no recurrence (D). Low PD-L1 and G6PD expression in a primary lesion of the same Case shown in figure 4F (E). Upregulated PD-L1 expression and stable low G6PD expression in the skin metastatic lesion of the same case shown in figure 4E (F).

G6PD can be used as a promising prognostic marker. Kaplan-Meier survival curves comparing MCC with high and low expression of G6PD classified based on RNA expression (counts per million; CPM) calculated by NGS showed significant differences in disease-specific survival (n=40, p=0.027, log-rank test, figure 4A). The cut-off value (1072 CPM) was calculated by the receiver operating characteristics curve. Immunohistochemical staining of G6PD using all FFPE samples, including primary and skin metastatic lesions, also correlated with patient outcome (n=79, p=0.034, log-rank test, figure 4B). Staining of more than 50% of tumor cells was defined as ‘G6PD high’. Representative samples of G6PD-high and G6PD low are shown in figure 4C, D. Both cases underwent spontaneous regression after biopsy. The case shown in figure 4C, however, experienced distant recurrence 10 months later and died after a few days, as we previously reported.^12^ The case shown in figure 4D had a good prognosis with no recurrence. These clinical outcomes might be predicted by the high expression of G6PD in the primary lesion. Immunohistochemical expression of G6PD has less heterogeneity than that of PD-L1. Even in the same case, expression of PD-L1 is heterogeneous, as we previously reported.^5 6^ In that case, PD-L1 expression was low in a primary lesion (figure 4F, left). Although multiple skin and lymph node metastases occurred, PD-L1 expression dramatically increased in a skin metastatic lesion (figure 4F, left) and eventually the patient had a good prognosis. Contrary to the changing PD-L1 expression, G6PD expression was consistently low in the primary and metastatic lesions (figure 4E and F, right). G6PD expression is a more useful predictor than PD-L1 expression and reflects the potential of using measures of immune activity as a prognostic factor.

### G6PD activity in blood serum sensitively reflects the clinical course

**Figure 5:**
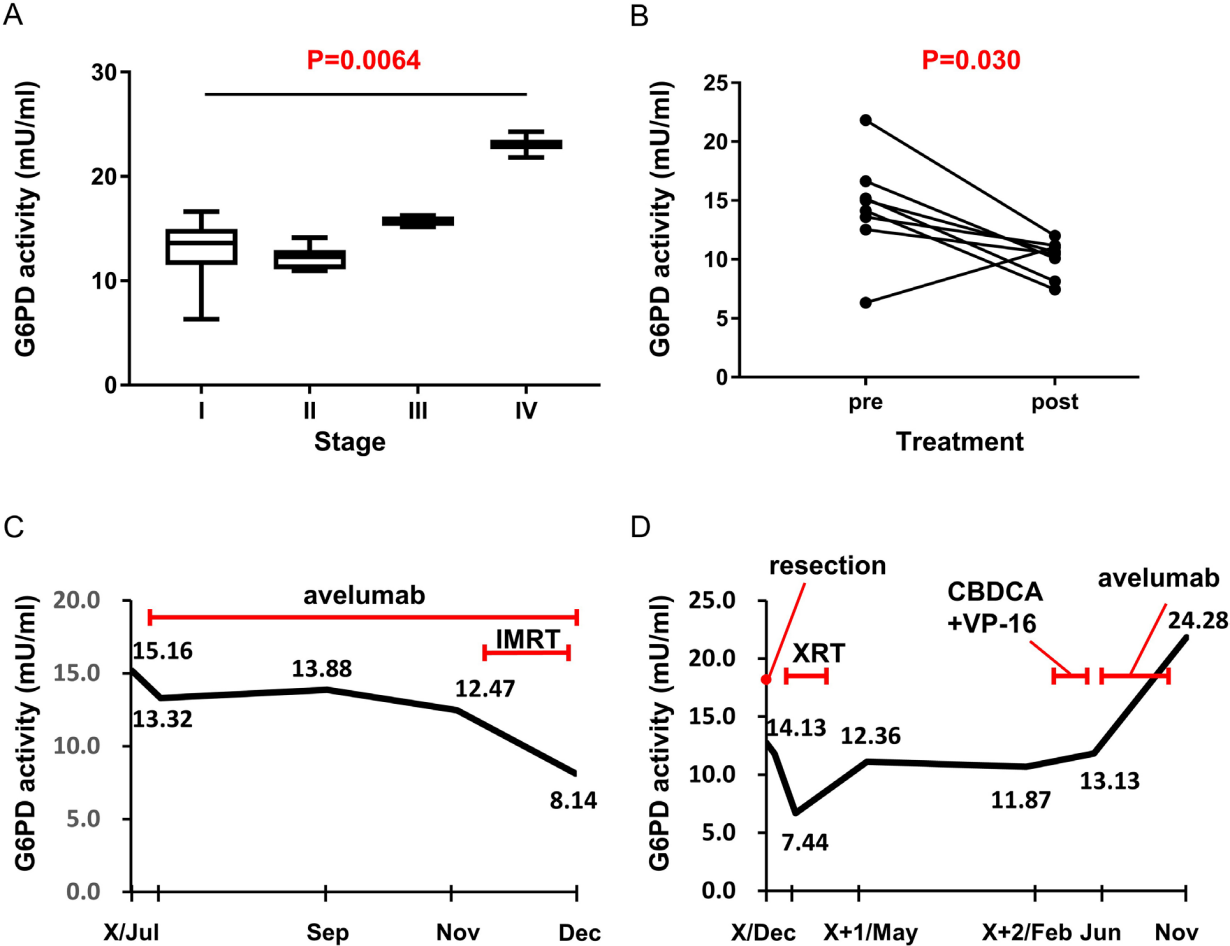
Serum G6PD activity significantly increased as the tumor stage progressed in samples obtained from pretreated patients (n=19, p=0.0064, Kruskal-Wallis test) (A). Serum G6PD activity decreased after treatment including surgery, radiation, or both (n=8 paired, p=0.030, paired-t test) (B). Representative data of the G6PD activity change in cases responding to avelumab. Serum G6PD activity decreased during treatment (C). Representative data of G6PD activity changes in cases not responding to avelumab. Serum G6PD activity increased during administration of the ICI (D). CBDCA, carboplatin; ICI, immune checkpoint inhibitor; IMRT, intensity modulated radiation therapy; VP-16, etoposide; XRT, X-ray radiation therap.

G6PD activity is measurable by a blood serum test. We collected 53 samples from 21 patients with MCC in various stages (summarized in table 2). In contrast to the immunohistochemical expression of G6PD, G6PD activity in the serum is constantly changing. Serum G6PD activity significantly increased as the tumor stage progressed in samples obtained from pretreated patients (n=19, p=0.0064, Kruskal-Wallis test, figure 5A) and decreased after treatment including surgery, radiation or both (n=8 paired, p=0.030, paired-t test, figure 5B). The change in G6PD activity in the two representative cases treated with the immune checkpoint inhibitor (ICI) avelumab is shown in figure 5C, D. The case shown in figure 5C responded to avelumab and the serum G6PD activity decreased during treatment. In contrast, the case shown in figure 5D did not respond to avelumab and the serum G6PD activity increased during administration of the ICI; the patient died a short while later. Serum G6PD activity may, therefore, be a useful tumor marker reflecting tumor progression and evaluating the response to treatment.

## Discussion

RNA sequence analysis of 395 cancer biomarkers revealed that MCC can be divided into two types: a ‘cell division type’ and an ‘immune active type’. G6PD expression seems to be a marker for distinguishing between these two types. G6PD can be used as a prognostic predictor and is detected not only by gene expression analysis and immunohistochemical staining, but also by serum tests. G6PD is a cytoplasmic enzyme and provides nicotinamide-adenine dinucleotide phosphate as a part of the pentose phosphate pathway. G6PD deficiency causes hereditary hemolytic anemia.^13^ Expression of G6PD is upregulated in numerous cancers, including esophageal squamous cell carcinoma,^14^ breast cancer,^15^ hepatocellular carcinoma,^16 17^ colon and colorectal cancer,^18 19^ renal cell carcinoma,^20^ bladder cancer^21^ and cervical cancer,^22^ and is associated with tumor cell proliferation, migration and invasion.^14–22^ To our knowledge, no studies have reported on the role of G6PD in MCC. The main role of G6PD in cancer cells is to protect against oxidative damage-induced cell death regulated by tumor suppressor gene P53,^23–25^ which is the most frequently mutated gene in MCC.^26^ High expression of G6PD leads to long survival of tumor cells and a poor prognosis. Therefore, G6PD is reported to be a prognostic marker.^27–29^ Additionally, our recent study of MCC demonstrated that G6PD is not only a prognostic predictor, but also a useful marker for judging the potential immune activity of a tumor. Low expression of G6PD indicates high immune activity and may suggest a good response for immune checkpoint blockade therapy. The mechanism of the linkage between G6PD and immune activity is still unclear. Because G6PD has a role to protect cells from cell death, we speculate that its downregulation may lead to oxidative stress-induced immunogenic cell death in cancer cells. Antigen presentation following immunogenic cell death will activate tumor immunity.

Immune checkpoint therapies, such as PD-L1 or PD-1 blockade therapy, for MCC, have recently shown successful results.^30 31^ Approximately 50% of patients, however, remain without durable benefit from these epochal treatments.^32^ Further immune checkpoint treatment strategies are therefore still required. The classification of MCC according to G6PD expression will provide new insight into the risk assessment and effect prediction of ICIs, including their usage as adjuvant or neoadjuvant therapies. G6PD blockade is a promising therapeutic option. G6PD inhibitors block the pentose phosphate pathway and inhibit cancer cell proliferation and metastases.^33^ Although G6PD inhibitors are reported to be effective against several cancer cells,^34 35^ their combination with G6PD blockade and ICIs has not been reported. According to our hypothesis, G6PD blockade will induce immunogenic cell death and enhance the efficacy of immunotherapy. However, it was reported that G6PD inhibition also suppresses the activity of immune cells including T cells.^36^ To activate tumor immunity, tumor-selective G6PD inhibition is desired.

MCC is an immune-sensitive tumor and will provide good study material for gaining an understanding of the mechanisms of tumor immunity and its evasion. Further studies of the actions of G6PD in MCC will facilitate the emergence of new therapeutic concepts for all malignancies.

## Conclusions

We found that G6PD is not only a useful prognostic marker, but also acts as an indicator to classify cases based on the tumor immune activity and a potential biomarker for immune checkpoint blockade therapy. G6PD can be detected by immunohistochemical staining and blood serum tests. Monitoring the effects of immunotherapy using simple methods will help to promote the benefits of these epochal treatments. The relationship between G6PD and immune activity in MCC opens up a new therapeutic concept for all malignancies.
