# Supplementary material for: Glucose-6-phosphate dehydrogenase correlates with tumor immune activity and programmed death ligand-1 expression in Merkel cell carcinoma
Source: J Immunother Cancer. 2020 Dec 23;8(2):e001679. doi: 10.1136/jitc-2020-001679 (PMC7759960; doi:10.1136/jitc-2020-001679)
Supplement: Supplementary data [file jitc-2020-001679supp001.pdf]

Table S1 Characteristics and treatment data for patients in immunohistochemical analysis

FFPE tissue

Characteristics		Value
cases		71
samples		90
Age(range)		77.27(40-98)
Sex		
	Male	26(36.6%)
	Female	45(63.4%)
Race		
	Asian(Japanese)	71(100%)
Primary Site		cases(n=71)
	Head&Neck	48(67.6%)
	Trunk	2(2.8%)
	Limbs	21(29.6%)
Lesion		samples(n=90)
	Primary	62(68.9%)
	Skin Meta	13(14.4%)
	Lymph Node	12(13.3%)
	Other	3(3.3%)
Stage at collection		samples(n=90)
	Ⅰ	26(28.9%)
	Ⅱ	24(26.7%)
	Ⅲ	28(31.1%)
	Ⅳ	9(10.0%)
	unknown	3(3.3%)
Treatment		cases(n=71)
	Surgery	16(22.5%)
	RT	5(7.0%)
	Surgery+RT	36(50.7%)
	Surgery+Chemo	2(2.8%)
	Surgery+RT+Chemo	5(7.0%
	Surgery+RT+ICI	2(2.8%)
	Observation	4(5.6%)
	Unknown	1(1.4%)
