# Supplementary material for: Glucose-6-phosphate dehydrogenase correlates with tumor immune activity and programmed death ligand-1 expression in Merkel cell carcinoma
Source: J Immunother Cancer. 2020 Dec 23;8(2):e001679. doi: 10.1136/jitc-2020-001679 (PMC7759960; doi:10.1136/jitc-2020-001679)
Supplement: Supplementary data [file jitc-2020-001679supp002.pdf]

Table S2 List of gene sets enriched in group A with a p-value less than 0.05 and in group B with a p-value less than 0.01.

Gene set	NES	p-value	FDR q-value
Group A (Nominal p-value < 5%)			
GO_CELL_DIVISION	1.630	0.047	1.000
Group B (Nominal p-value < 1%)			
GO_T_CELL_RECEPTOR_SIGNALING_PATHWAY	-1.767	0.000	1.000
GO_TYPE_2_IMMUNE_RESPONSE	-1.681	0.004	1.000
GO_REGULATION_OF_IMMUNE_RESPONSE	-1.656	0.002	1.000
GO_ANTIGEN_RECEPTOR_MEDIATED_SIGNALING_PATHWAY	-1.618	0.000	1.000
GO_INTERFERON_GAMMA_PRODUCTION	-1.607	0.004	1.000
GO_REGULATION_OF_IMMUNE_SYSTEM_PROCESS	-1.601	0.008	0.992
GO_EXTERNAL_SIDE_OF_PLASMA_MEMBRANE	-1.530	0.006	0.926
