# Supplementary material for: Glucose-6-phosphate dehydrogenase correlates with tumor immune activity and programmed death ligand-1 expression in Merkel cell carcinoma
Source: J Immunother Cancer. 2020 Dec 23;8(2):e001679. doi: 10.1136/jitc-2020-001679 (PMC7759960; doi:10.1136/jitc-2020-001679)
Supplement: Supplementary data [file jitc-2020-001679supp003.pdf]

Table S3 Fisher's exact tests revealed a significant association between groups A and B with regard to PD-L1 expression in MCC cells.

Fisher's exact test

Group A vs. B	Significance (p)	OR (95% CI)
MCPyV - vs. +	0.33	2.1 (0.47-9.44)
Regression - vs. +	0.34	3.2 (0.30-34.64)
Metastasis - vs. +	0.17	0.38 (0.095-1.53)
PD-L1 Low vs. High	0.032*	7.0 (1.18-41.36)
